# Plant mitochondrial PORR proteins facilitate group II intron splicing by binding to distinct regions within their target introns

**DOI:** 10.1093/nar/gkaf781

**Published:** 2025-08-18

**Authors:** Chuande Wang, Martine Quadrado, Talla Ngom, Hakim Mireau

**Affiliations:** School of Agriculture and Biology, Joint Center for Single Cell Biology/Shanghai Collaborative Innovation Center of Agri-Seeds, Shanghai Jiao Tong University, Shanghai, 200240, China; Université Paris-Saclay, INRAE, AgroParisTech, Institute Jean-Pierre Bourgin for Plant Sciences (IJPB), 78000 Versailles, France; Université Paris-Saclay, INRAE, AgroParisTech, Institute Jean-Pierre Bourgin for Plant Sciences (IJPB), 78000 Versailles, France; Université Paris-Saclay, INRAE, AgroParisTech, Institute Jean-Pierre Bourgin for Plant Sciences (IJPB), 78000 Versailles, France

## Abstract

Ribozymic introns are widely distributed in eubacteria and organelles such as mitochondria and chloroplasts. In plants, organellar introns often exhibit degenerate RNA structures, lacking essential elements for self-splicing and mobility. Consequently, their splicing relies heavily on host-encoded proteins. The plant organellar RNA recognition (PORR) domain, a recently identified RNA-binding motif, defines a small, plant-specific protein family. In this study, we characterized five novel mitochondria-targeted PORR genes. Functional analyses revealed that all five PORRs play a role in mitochondrial group II intron splicing. While one PORR protein was found necessary for the splicing of a single intron, the other four facilitate the splicing of multiple introns. Splicing defects in each *porr* mutant resulted in a significant reduction in complex I assembly and activity, along with an increase in the levels of other respiratory complexes. *In vivo* RNA binding studies revealed that the analyzed PORR proteins bind either to the 3′ end of trans-spliced intron 5′-halves or within intron domain I, indicating potential roles in trans-spliced intron rejoining or in the structural organization of domain I. Overall, these findings demonstrate that nuclear-encoded PORR proteins play crucial roles in the splicing of specific mitochondrial group II introns and are essential for mitochondrial biogenesis.

## Graphical Abstract

**Graphical Abstract:**
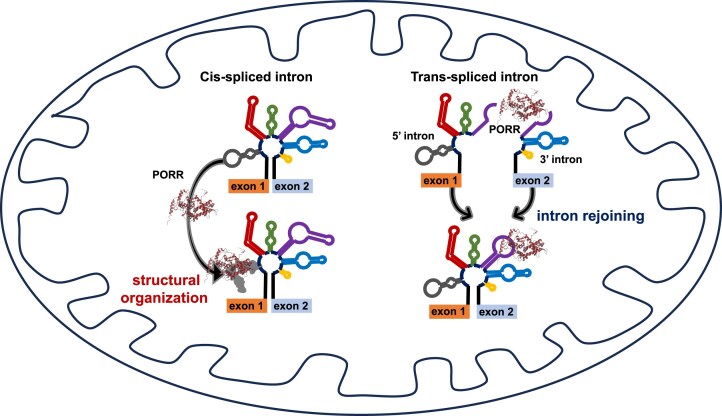

## Introduction

Mitochondria are essential eukaryotic organelles that produce most of cellular energy through oxidative phosphorylation. In addition to energy production, they play crucial roles in various cellular processes, including amino acid and nucleotide metabolism, apoptosis regulation, ion transport, and hormone signaling in plants [1]. These organelles have an exogenous origin and are thought to have arisen from the endosymbiotic association of a bacteria belonging to a sister group of the alpha-proteobacteria [2]. The transition from original endosymbionts to permanent organelles was accompanied by a massive reduction in their genetic content and a progressive loss of autonomy [3, 4]. Modern mitochondrial genomes have retained a limited number of bacteria-derived genes that are still expressed within the organelle. A vast cohort of host-derived proteins, either acquired or recruited during evolution, is thus essential for proper expression of mitochondrial genes. This cooperation between highly degenerated bacterial-like infrastructures and eukaryotic-derived functions resulted in a mitochondrial RNA metabolism that is far more complex than that of modern bacteria [5]. Additionally, gene expression in plant mitochondria is primarily controlled at the post-transcriptional levels [5]. One of the most remarkable features of plant mitochondrial messenger RNA (mRNA) expression involves the splicing of numerous introns that interrupt protein-coding genes and whose elimination from transcripts is required to produce most mitochondria-encoded proteins [5–8]. According to their structures and splicing reactions, mitochondrial introns could be classified in two families—named group I and group II—and most seed plant mitochondrial introns belong to the group II [6, 7]. Interestingly, while most mitochondrial introns consist of a single continuous sequence, some are split into two distinct segments, each carried by independently transcribed precursor RNAs. These intron halves are subsequently joined through a process known as trans-splicing, resulting in a single mature transcript [7]. Despite little sequence conservation, group II introns adopt a conserved secondary structure consisting of six double-helical domains (D I to D VI) [9–12]. Unlike their autocatalytic progenitor, plant mitochondrial introns are highly degenerate and have lost the capacity of self-splicing. Many protein cofactors encoded either by nuclear or the mitochondrial genome are therefore required for intron splicing. Some of these factors, such as maturase-related proteins (e.g. MatR and its four nuclear-encoded homologs nMAT1 to nMAT4), originate from the endosymbiont [13–19]. However, most other splicing factors have arisen through co-evolution between the nuclear and mitochondrial genomes. These factors belong to different protein families like pentatricopeptide repeat (PPR) proteins, chloroplast RNA splicing and ribosome maturation (CRM) proteins, plant organellar RNA recognition (PORR) proteins, mitochondrial transcription termination factor (mTERF), and uL18 ribosomal proteins [20–22]. Factors like maturases, RNA helicases, and certain CRM proteins are required for the splicing of several mitochondrial group II introns. Other factors appear to be specific to individual or a small subset of group II introns. However, the mechanisms by which these factors facilitate splicing remain unclear, and many protein factors involved in the process likely still remain unidentified.

Unlike PPR proteins, which have been extensively studied, the mechanisms by which splicing factors interact with RNA sequences remain elusive. Proteins such as mTERFs, which are composed of repeated motifs, may bind RNA in a sequence-specific manner similar to PPR proteins. In this study, we investigated the poorly characterized PORR protein family, which, in contrast to PPR and mTERF proteins, lacks repeated motifs and may instead recognize structured RNA domains rather than linear sequence elements. We characterized the function of five novel mitochondria-targeted PORR proteins in Arabidopsis, designed as PORR5, PORR8, PORR10, PORR11, and PORR14. We demonstrate that these PORR proteins are essential for the splicing of one or multiple group II introns in transcripts encoding respiratory complex I subunits and that they specifically associate with distinct structural domains within their target intron *in vivo*. The loss of PORR proteins leads to defects in complex I biogenesis, the activation of the alternative respiratory pathway, and impaired plant growth.

## Materials and methods

### Plant material

Arabidopsis (*Arabidopsis thaliana*) Col-0 plants were obtained from the Arabidopsis stock centre of the Institut National de Recherche pour l′Agriculture, l′Alimentation et l′Environnement in Versailles (http://dbsgap.versailles.inra.fr/portail/). The Arabidopsis T-DNA mutants N816884 (*porr5-1*), N822100 (*porr8-1*), N526531 (*porr8-2*), N570727 (*porr10-1*), N530315 (*porr10-2*), N545217 (*porr11-1*), and N530388 (*porr14-1*) were acquired from the European Arabidopsis Stock Centre (http://arabidopsis.info/). Homozygous mutants for the insertions were identified by PCR genotyping using specific primers (Supplementary Table S2). Plants were grown on soil in a greenhouse under long-day conditions (16 h of light and 8 h of dark).

The PSB-D cells [23] are a suspension culture of *A. thaliana* (ecotype Landsberg erecta), kindly provided by Geert De Jaeger (Ghent University, Ghent, Belgium). They were cultured and transformed as previously described (https://abrc.osu.edu/researchers/cell).

### Functional complementation of the T-DNA mutants

For plant complementation analysis of *porr5*, *porr11*, and *porr14* mutants, the full-length coding sequences and native gene promoter regions (1000 bp upstream of ATG site) of the corresponding PORR were amplified by PCR, cloned into pDONR207 vector by Gateway BP reaction (Invitrogen), and subsequently transferred into the pGWB13 binary vector [24]. The resulting constructs (PORR_pro_::PORR-3HA) were transformed into *Agrobacterium tumefaciens* C58C51 and used for floral dip transformation in the corresponding *porr* heterozygous plants. Functionally complemented homozygous mutant plants were identified in the progenies of transformed plants by PCR analysis.

### Subcellular distributions

The full-length coding regions of each PORR protein, but lacking their stop codon, were PCR amplified and transferred into pGWB5 [24] by Gateway cloning (Invitrogen) to create GFP translational fusions. The fusion constructs were transformed into the PSB-D Arabidopsis cell culture as indicated in [23]. GFP fluorescence was visualized in transgenic cell lines by Leica TCS SP5 confocal microscopy. Prior to observation, cell culture cells were soaked in 0.1 μM Mitotracker Red (Invitrogen) to label mitochondria.

### Blue native gel and in-gel activity assays

Crude Arabidopsis mitochondrial extracts from 8-week-old flower buds were prepared as previously described [25]. An equivalent to 120 mg of mitochondrial proteins was loaded and separated on 4%–16% (w/v) polyacrylamide NativePAGE Bis/Tris gels (Invitrogen). After electrophoresis, BN-PAGE gels were either subjected to in-gel activity assay or transferred to polyvinylidene difluoride (PVDF) membranes as previously described in [25]. Membranes were then hybridized with specific antibodies (Supplementary Table S3). Hybridization signals were detected by using enhanced chemiluminescence reagents (Western Lightning Plus ECL, Perkin Elmer).

### Protein immunodetection

Total proteins were extracted from crude membrane and separated by sodium dodecyl sulphate–polyacrylamide gel electrophoresis (SDS–PAGE). Gels were then transferred to PVDF membranes and incubated with antibodies and dilutions indicated in Supplementary Table S3.

### RNA extraction and analysis

Total RNA was isolated from 8-week-old flower buds using TRIzol reagent (Life Technologies) according to the manufacturer’s instructions. RNA was treated with DNase Max (QIAGEN) when RNAs were used in reverse transcription and PCR assays [e.g. RT-PCR or RT-quantitative PCR (RT-qPCR)]. First-strand complementary DNA (cDNA) synthesis was performed on 1 μg of RNA using M-MLV Reverse Transcriptase 1st-Strand cDNA Synthesis Kit (Invitrogen). For expression analysis of each *PORR* gene, RT-PCR analysis was performed by using primer pairs listed in Supplementary Table S2. The *BIO2* gene was used as a control to check cDNA integrity.

Mitochondrial transcriptome analysis and quantification of splicing efficiency of mitochondrial introns were performed by RT-qPCR as previously described [26]. In short, specific primer pairs were used for each mitochondrial intron to quantify spliced or precursor transcripts in both wild-type and mutant plants. After normalization across replicates, the ratios of precursor to mature transcripts were calculated to evaluate the splicing efficiency of each intron. Finally, mutant-to-wild-type splicing efficiency ratios were determined to identify potential splicing defects in the mutant lines. Quantitative RT-PCR was performed using SsoAdvanced Universal SYBR Green Supermix (Bio-Rad) and a Bio-Rad CFX96 real-time PCR detection system with the following thermal cycling program: 95°C for 3 min, followed by 39 cycles of 95°C for 5 s, 60°C for 20 s, and 72°C for 10 s. Each reaction contained 5 μl SYBR Green Supermix, 0.5 μl of each primer (10 μM), 2 μl of diluted cDNA, and nuclease-free water to a total volume of 10 μl. No-template and RT-minus controls were included to ensure the absence of contamination. Two biological and three technical repeats were performed and the nuclear 18S ribosomal RNA gene was used for data normalization across samples.

RNA gel blot analyses were performed following the general procedure described in [27], with the key steps detailed below to ensure reproducibility. Briefly, 15 μg of total RNA was separated on a 1.5% agarose gel containing 3% formaldehyde in 1 × MOPS buffer [20 mM 3-(N-morpholino)propanesulfonic acid (MOPS), pH 7.0, 5 mM sodium acetate, 1 mM ethylenediaminetetraacetic acid (EDTA)]. After electrophoresis at 100 V for 4 h, RNA was transferred to a GeneScreen nylon membrane (PerkinElmer) via capillary transfer using 10 × saline-sodium citrate (SSC) buffer and crosslinked by UV irradiation (Stratalinker, Stratagene). Hybridizations were carried out overnight at 65°C in Church buffer [7% SDS, 0.25 M Na_2_HPO_4_ (pH 7.4), 2 mM EDTA, 200 μg/ml heparin] using [α-32P]-labeled probes generated by random priming. Blots were washed twice in 1 × SSC, 0.1% SDS, and once in 0.1 × SSC, 0.1% SDS at 65°C, then exposed to a phosphorimager screen and scanned with a Typhoon FLA 9500 system (GE Healthcare). The DNA fragments used for probe synthesis were PCR-amplified using gene-specific primers listed in Supplementary Table S2.

### RNA immunoprecipitation assays

RIP assays were performed with the μMACS GFP-Tagged Protein Isolation Kit (Miltenyi Biotec) according to the manufacturer’s instructions but with minor modifications. Cells from 3-day-old diluted Arabidopsis cultures expressing 35Spro::PORR-GFP fusions were collected and ground to a fine powder under liquid nitrogen using a mortar and pestle. Samples were homogenized in cold lysis buffer [20 mM HEPES-KOH, pH 7.6, 100 mM KCl, 20 mM MgCl_2_, 1 mM Dithiothreitol (DTT), 1% Triton X-100, 1× of complete EDTA-free protease inhibitor (Roche)] for 30 min on ice with slow rotation. The lysate was centrifuged at 100 000 × *g* for 20 min at 4°C. The clarified supernatant was incubated with 50 μl of anti-GFP magnetic beads (Miltenyi Biotec) for 1 h at 4°C with rotation (10 rpm) and then loaded on μMACS column. Following three washes with 1 ml of washing buffer (lysis buffer with only 0.1% Triton X-100), immunoprecipitated proteins were eluted using 90 μl of elution buffer (Miltenyi Biotec) and subjected to protein analysis. To recover co-immunoprecipitated RNAs, beads were eluted with 1000 μl of TRI Reagent^®^ (Invitrogen). Prior to RT-qPCR analysis, immunoprecipitated RNA was treated with DNase Max (QIAGEN). RNAs representing 1% of the input fraction and the totality of immunoprecipitated RNAs were used in RT-qPCR analysis. Two biological and three technical replicates were performed, and the untransformed cells (PSB-D) were used as negative control.

### Accession numbers

Sequence data from this article can be found in the GenBank/EMBL data libraries under accession numbers RPD1, AT4G33495; PORR5, AT5G45790; PORR8, AT2G31290; PORR10, AT4G08940; PORR11, AT3G63090; and PORR14, AT1G06440.

## Results

### PORR proteins are essential for normal plant growth and development

**Figure 1.**
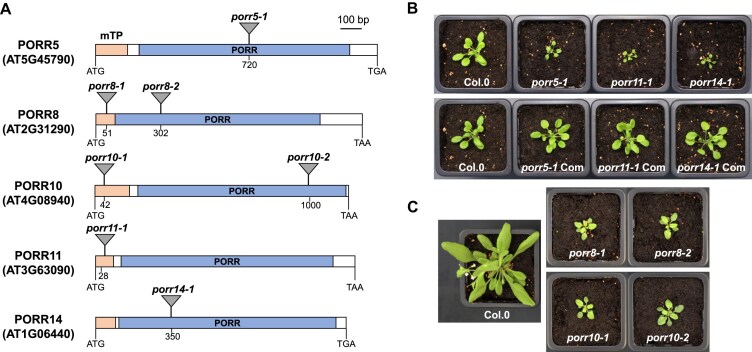
Developmental delays in Arabidopsis *porr* mutants. (**A**) Schematic representation of the five analyzed *PORR* genes (*PORR5*,*PORR8*,*PORR10*,*PORR11*,and*PORR14*). The locations of T-DNA insertion sites within each gene are indicated. The putative mitochondrial transit peptideand PORR domain coding regions are shown as orange and blue boxes, respectively. (**B**) Photograph of 4-week-old plants showing the reduced size of *porr5-1*, *porr11-1*,and*porr14-1* homozygous mutants compared with wild-type (Col-0) and genetically complemented (Com) plants. (**C**) Photograph of 6-week-old plants illustrating the slow-growth phenotype of *porr8* and *porr10* homozygous mutants compared to a wild-type plant.

The PORR proteins share a recently recognized RNA-binding domain and form a small plant-specific protein family [28–30]. The PORR gene family comprises 15 members in *A. thaliana*, two of them are predicted to localize in plastids and 13 in mitochondria. Three members of this protein family have been convincingly shown to be involved in organellar intron splicing [28–30]. To get insight into the function(s) of other mitochondria-targeted PORR proteins, we screened available T-DNA insertion lines in *PORR* genes and identified five mutants exhibiting overall growth retardation compared to wild-type plants (Fig. 1). The concerned PORR genes corresponded to *AT5G45790* (*PORR5*), *AT2G31290* (*PORR8*), *AT4G08940* (*PORR10*), *AT3G63090* (*PORR11*), and *AT1G06440* (*PORR14*). Two independent T-DNA lines were identified for *PORR8* and *PORR10*, and both mutant lines showed very similar phenotypes. Single mutants were isolated for *PORR5*, *PORR11*, and *PORR14*, and the observed mutant phenotypes in these lines were fully complemented by introducing an HA-tagged copy of the corresponding *PORR* gene into the respective mutants. PCR analysis and sequencing confirmed that the T-DNA insertions were located either in the mitochondrial targeting N-terminal presequence or within the PORR domain coding regions (Fig. 1). RT-PCR analysis failed to detect full-length mRNAs derived from each *PORR* gene in the respective mutants, strongly suggesting that the identified mutant alleles correspond to null mutations (Supplementary Fig. S1). Together, these results indicate that the observed developmental alterations were due to the disruption of the concerned *PORR* genes.

### Selected PORR proteins localize to the mitochondria

**Figure 2.**
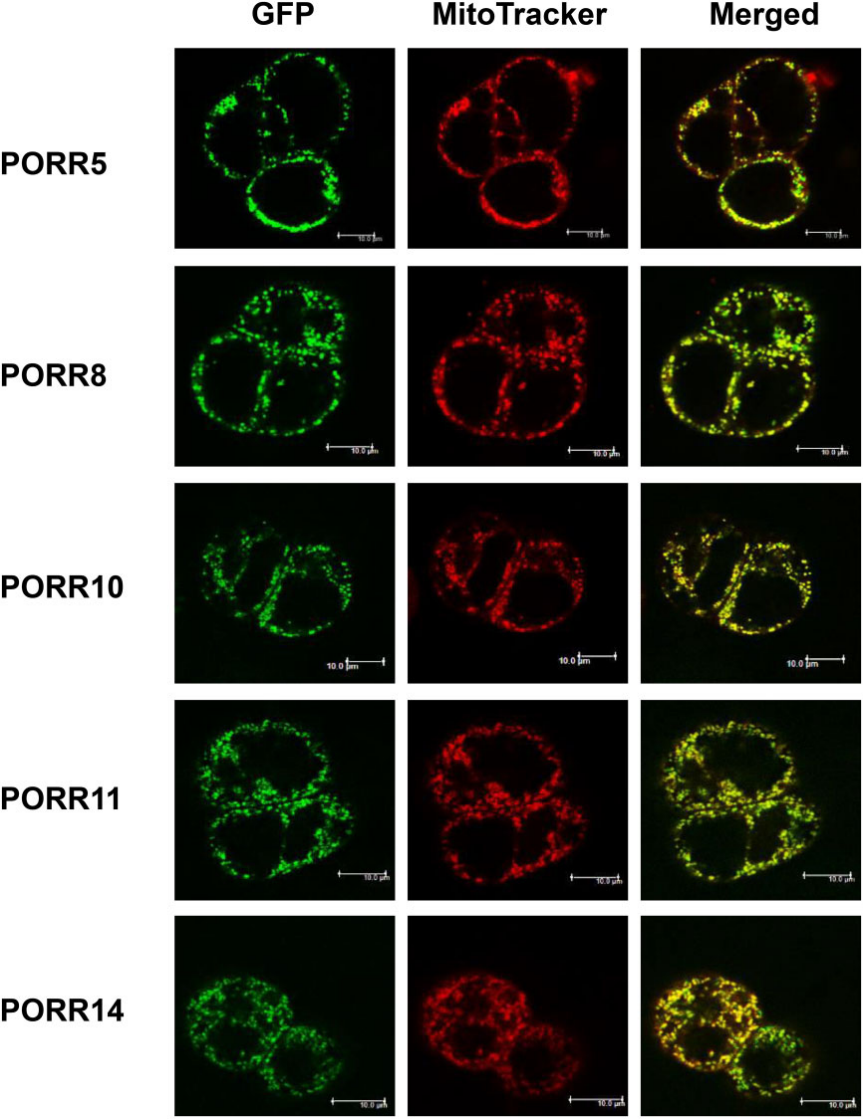
The Arabidopsis PORR proteins are transported into mitochondria. Confocal microscope images showing the subcellular distribution of PORR-GFP translational fusions in transgenic Arabidopsis cell lines. The left panels show GFP fluorescence, the center shows mitochondria labeled with the MitoTracker Red dye, and the right panels present the merged signals. Bar = 10 μm.

Based on the Arabidopsis subcellular database SUBA (http://suba.live/), PORR5, PORR8, PORR10, and PORR14 proteins were all predicted to contain a putative mitochondrial localization signal in their N-terminal region. The only exception is the PORR11 protein, which was predicted to localize to cytosol. To validate these predictions, C-terminal GFP translational fusions comprising the full length of each PORR protein were expressed into *in vitro*-grown Arabidopsis PSB-D cells [23]. Cells from transgenic lines were analyzed by confocal microscopy, which revealed that the PORR-GFP signals concentrated in small cellular patches. In all cases, the GFP fluorescence colocalized with MitoTracker red signals (Fig. 2), providing experimental evidence that the five analyzed PORR proteins are localized to mitochondria.

### PORR genes are required for the splicing of distinct mitochondrial introns

**Figure 3.**
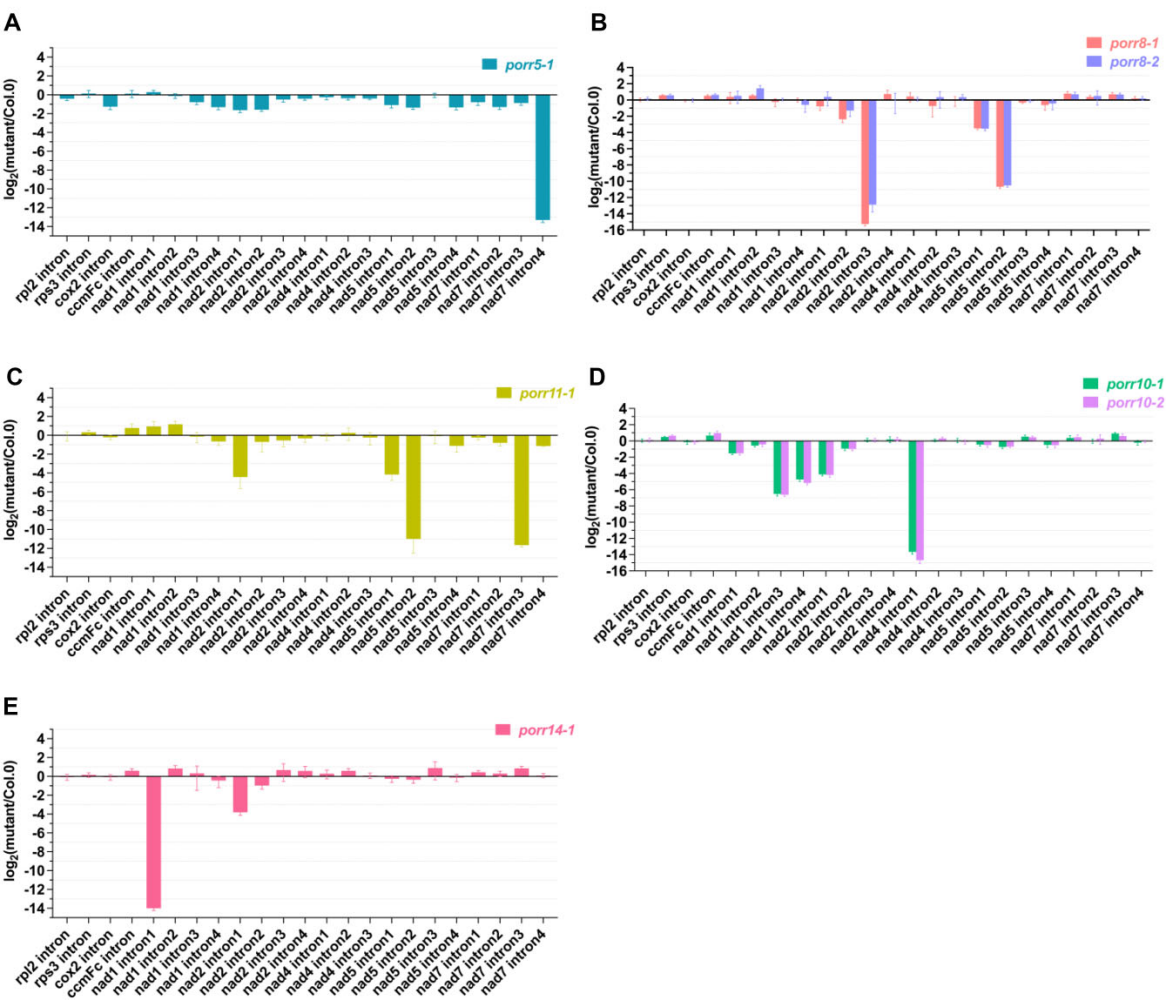
The splicing efficiencies of various mitochondrial introns are decreased in *porr* mutants. RT-qPCR analysis of splicing efficiency for all 23 introns in Arabidopsis mitochondrial transcripts (see “Materials and methods” section for details). Bars represent log_2_ ratios of splicing efficiencies in *porr* plants to the wild type (Col-0). Three technical replicates and two independent biological replicates were used for each genotype; standard errors are indicated. (**A**–**E**) show the results for the *porr5*, *porr8*, *porr11*, *porr10*, and *porr14* mutants, respectively.

**Figure 4.**
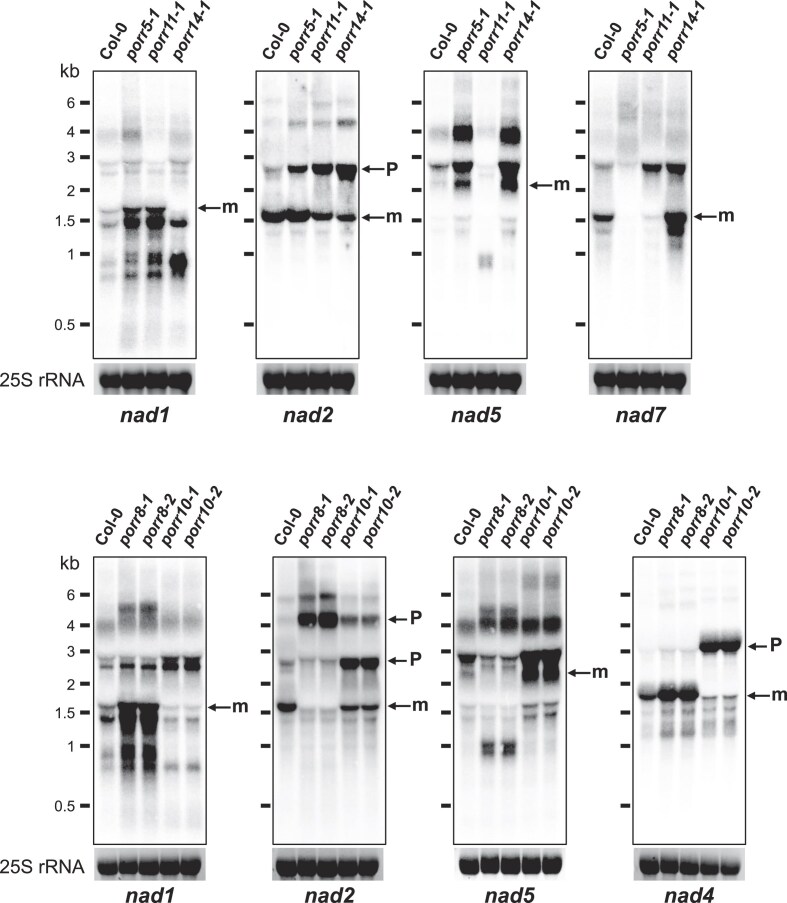
RNA gel blot analyses showing the accumulation profiles of affected complex I (*nad*) transcripts in the five *porr* mutants. Total RNA (15 μg) was prepared from *porr* mutants and wild-type (Col-0) flowers and hybridized with the indicated probes. Signals corresponding to the expected mature transcripts are indicated by “m” and precursors by “P.” Ethidium bromide staining of ribosomal RNAs is shown below the blots and serves as a loading control. RNA marker sizes are indicated (kb).

Previous studies have shown that PORR proteins function in group II intron splicing in chloroplasts or mitochondria [28–30], suggesting that the five PORR proteins described here may also play key roles in mitochondrial RNA metabolism. To test this hypothesis, we measured the steady-state levels of both precursor and mature mitochondrial transcripts by RT-qPCR and compared the splicing efficiencies of mitochondrial introns in the five *porr* mutants with those in wild-type plants (Fig. 3 and Supplementary Fig. S2). The majority of mature mitochondrial mRNAs accumulated at the same or slightly higher levels in all *porr* lines compared to Col-0 plants. However, we observed that the levels of specific regions of mature transcripts encoding complex I subunits were decreased to varying extents in certain mutants (Supplementary Fig. S2). The most significant alterations were observed in *nad7* mRNA in *porr5*; *nad2* and *nad5* mRNAs in *porr8*; *nad1*, *nad2*, and *nad4* mRNAs in *porr10*; *nad2*, *nad5*, and *nad7* mRNAs in *porr11*; and *nad1* and *nad2* mRNAs in *porr14* mutants (Supplementary Fig. S2). We further determined the splicing efficiencies of all 23 Arabidopsis mitochondrial introns in wild-type and mutant lines. These analyses revealed a pronounced reduction in splicing efficiency for *nad7* intron 4 in the *porr5* mutant (Fig. 3A). Meanwhile, several splicing defects were observed in the other four *porr* mutants. These included two major splicing reductions for *nad2* intron 3 and *nad5* intron 2, and a minor reduction for *nad5* intron 1 in *porr8* mutants (Fig. 3B). Similarly, a notable decrease in the splicing of *nad5* intron 2 and *nad7* intron 3, and milder reductions in *nad2* intron 1 and *nad5* intron 1 splicing were detected in *porr11* mutants (Fig. 3C). In *porr10* and *porr14* plants, a pronounced decrease in the splicing efficiency of one intron was observed, along with more minor splicing defects for one or more other introns. In *porr10* mutants, the splicing of *nad4* intron 1 was strongly affected, while *nad1* intron 3 and intron 4, as well as *nad2* intron 1 were much less affected (Fig. 3D). Similarly, a strong decrease in the splicing efficiency of *nad1* intron 1 and a slight reduction in *nad2* intron 1 splicing were detected in *porr14* plants (Fig. 3E). These findings were further supported by RNA gel blot analysis (Fig. 4), which confirmed the reduction in detectable mature mRNAs for the affected *nad* genes in the respective *porr* mutants. As a result, the corresponding unspliced transcripts accumulated to higher levels in the mutant lines (Fig. 4). Altogether, our results indicate that PORR proteins are indispensable for the splicing of various mitochondrial group II introns. While PORR5 is exclusively required for a single splicing event, PORR8, PORR10, PORR11, and PORR14 are necessary for the excision of two to four introns.

### Complex I biogenesis is strongly impaired in five *porr* mutants

**Figure 5.**
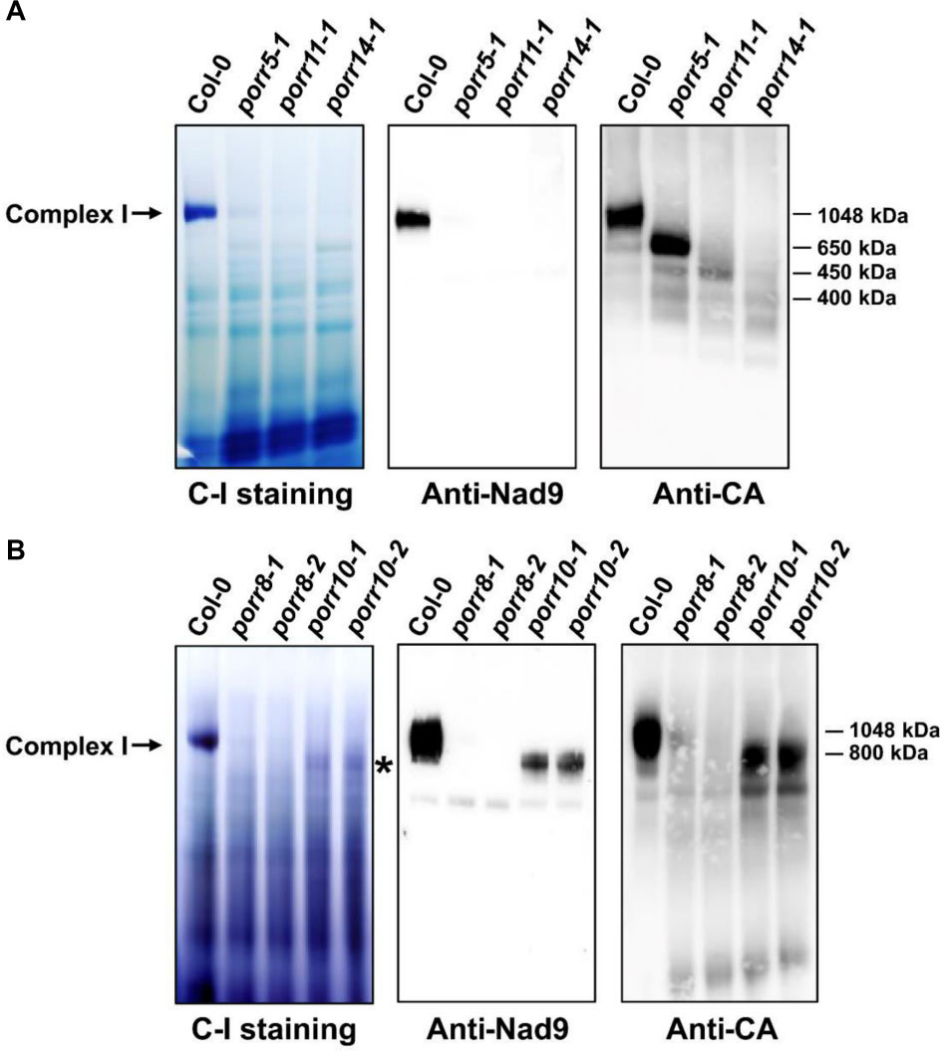
Impaired complex I biogenesis in *porr* mutants. Crude mitochondrial extracts from wild-type and *porr* mutants were separated on BN-PAGE gels. The left panels (C-I staining) show in-gel staining for NADH dehydrogenase activity of complex I. Following migration, BN-PAGE gels were transferred to membranes, and blots were probed with antibodies to mitochondrial Nad9 (center panel) and carbonic anhydrase (CA) subunit (right panel). Apparent molecular weights are indicated on the side. Panels (**A**) and (**B**) display results for *porr5*, *porr11*, *porr14*, *porr8*, and *porr10* mutants, respectively. Holocomplex I is indicated by an arrowhead, while the 800 kDa assembly intermediates in *porr10* mutants are shown by an asterisk.

The mitochondrial localization of the five PORR proteins led us to consider that the developmental abnormalities observed in *porr* mutants could result from mitochondrial respiratory dysfunctions. To investigate potential respiratory impairment, crude mitochondrial proteins were resolved by blue native PAGE, followed by in gel activity assays and immunodetection of respiratory chain complexes. The results showed that the accumulation of monomeric complex I were substantially decreased in all *porr* mutants compared with the wild type (Fig. 5). Immunodetection using antibodies against two complex I subunits (Nad9 and carbonic anhydrase) revealed the absence of the ∼1000 kDa complex I band in the mutants, which was clearly visible in wild-type plants. Instead, the accumulation of low-molecular-weight particles (around 650 kDa in *porr5*, 450 kDa in *porr11*, and 800 kDa in *porr10*), corresponding to complex I assembly intermediates [31], was observed in the mutants (Fig. 5). In-gel staining of complex IV and immunodetection with antibodies against respiratory chain subunits of complex III (RISP) and complex V (ATP synthase-β) showed an equal or higher abundance of these respiratory complexes in all *porr* lines compared to the wild type (Supplementary Fig. S3A and C). Furthermore, the abundance and activity of complex I were restored to Col-0 levels in the functionally complemented *porr5*,*porr11*, and *porr14* plants (Supplementary Fig. S3B).

**Figure 6.**
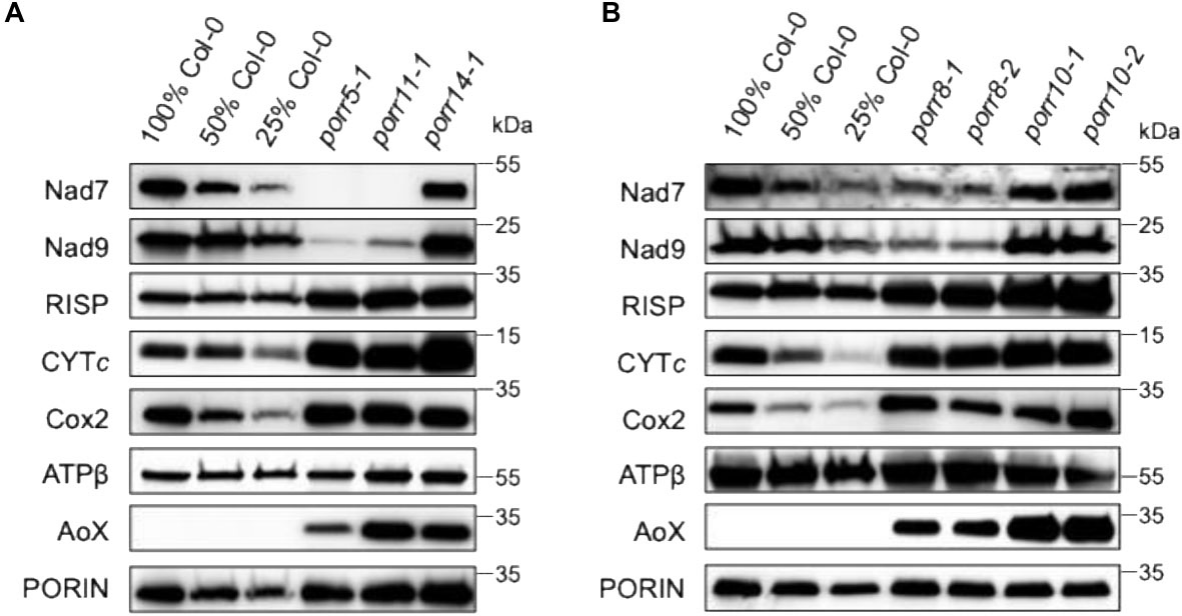
Immunoblot analysis of selected core subunits of respiratory chain complexes in *porr* mutants. Crude membrane extracts [∼50 μg from wild-type (100%) and mutant samples] were separated by SDS–PAGE and probed with antibodies to subunits of respiratory complex I (Nad7 and Nad9), complex III (RISP), complex IV (Cox2), the ATP synthase (ATPβ), cytochrome *c*, and the alternative oxidase (AOX). Immunoblots of Porin served as protein loading controls. Panels (**A**) and (**B**) show the results for the *porr5*, *porr11*, *porr14*, *porr8*, and *porr10* mutants, respectively.

We next analyzed the steady-state levels of constituent subunits of mitochondrial respiratory complexes by immunoblot analysis (Fig. 6). The abundance of RISP (complex III), cytochrome *c*, Cox2 (complex IV), and ATP-β (complex V) proteins was higher or close to normal levels in the mutants compared to wild-type (Fig. 6). These observations are consistent with the over-accumulation of complexes III and IV, but not complex V, as observed by BN-PAGE gel analysis (Supplementary Fig. S3). The accumulation of Nad7, whose corresponding transcript exhibited impaired intron splicing in *porr5* and *porr11* plants (Fig. 3), was accordingly greatly diminished in these two mutants. Interestingly, the accumulation of the Nad9 protein was also significantly reduced in *porr5* and *porr11* mutant plants (Fig. 6A). Moreover, in both *porr8* lines, Nad7 and Nad9 proteins accumulated to roughly 25% of wild-type levels (Fig. 6B), although the intron splicing of *nad7* mRNA was unaffected in these mutants (Fig. 3). In contrast, the levels of Nad7 and Nad9 proteins in *porr14* and *porr10* plants were indistinguishable from those in the wild type.

To further investigate the respiratory chain organization in the *porr* mutants, the expression levels of the AOX and alternative NADH dehydrogenase (*NDA, NDB*, and *NDC*) genes were assessed by RT-qPCR analysis. This approach revealed significant overexpression of several components of alternative respiratory pathways in most *porr* mutants, particularly the *AOX1A*, *AOX1D*, and *NDB4* genes (Supplementary Fig. S4). Accordingly, a dramatic increase in the abundance of the AOX proteins was detected by immunoblot analysis in all *porr* plants (Fig. 6).

Overall, these results led us to conclude that the biogenesis and activity of mitochondrial complex I were severely compromised in the analyzed *porr* mutants. The decreased splicing efficiency of distinct *nad* transcripts is thought to be responsible for the complex I deficiencies detected in these plants.

### PORR proteins are found in large RNA-containing particles and associate *in vivo* with their intron RNA targets

**Figure 7.**
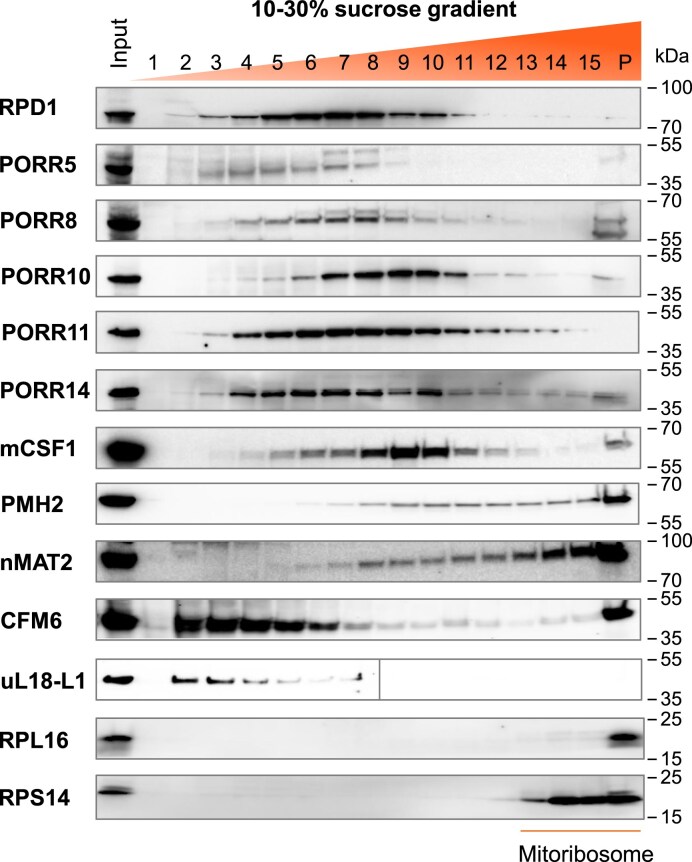
PORR proteins are found in high-molecular-weight complexes. Protein extracts from Arabidopsis cell lines overexpressing the indicated HA-tagged PORR proteins were fractionated on 10%–30% sucrose gradients, alongside extracts expressing control poly-acting splicing factors (mCSF1, PMH2, and nMAT2) and mono-acting splicing factors (CFM6 and uL18-L1). Equal volumes from each fraction of the gradient were analyzed by immunoblotting with an anti-HA antibody. The mitochondrial ribosomal proteins RPL16 and RPS14 served as markers to indicate the positions of mitochondrial ribosomes within the gradient. The sedimentation profile of uL18-L1 was previously reported [22].

Previously identified splicing factors for organellar group II introns have been found to associate with large intron-containing complexes [32, 33]. Similarly, the PORR-domain-containing protein WTF1 was detected in particles of a similar size range to these group II intron ribonucleoprotein complexes [29]. To investigate whether other PORR proteins are parts of large RNA-containing particles, we performed sucrose gradient centrifugation on extracts from Arabidopsis PSB-D cells [23] expressing tagged versions of the five PORR proteins analyzed in this study, along with RPD1, a PORR protein previously demonstrated to facilitate the splicing of multiple mitochondrial introns [30]. The resulting fractions were analyzed by immunodetection to determine the sedimentation profiles of each PORR protein. In line with the previously characterized PORR protein WTF1, the five PORR proteins and RPD1 were found in complexes of varying sizes, but significantly smaller than mitoribosomes (Fig. 7). Their sedimentation profiles were compared to those of control proteins involved in the splicing of either single introns (e.g. CFM6 [34] and uL18-L1 [22]) or multiple introns (e.g. mCSF1[35], PMH2 [18], and nMAT2 [16]). Our results revealed that mono-acting PORR proteins (i.e. those required for the splicing of a single intron) were predominantly found in the lighter sucrose gradient fractions (1 to 6), whereas poly-acting PORR proteins (those involved in the splicing of more than one intron) were enriched in the heavier fractions (9 to 15; see Fig. 7). The sedimentation profiles of PORR8, PORR10, and PORR11 closely resembled those of control poly-acting splicing factors, consistent with their role in the splicing of multiple introns. In contrast, PORR5, which is involved in the splicing of a single intron, exhibited a distribution pattern similar to that of the mono-acting factors CFM6 and uL18-L1. Interestingly, PORR14 displayed an intermediate distribution profile, sharing characteristics of both mono- and poly-acting splicing factors, suggesting a role that does not fit strictly within either category. These distinct sedimentation patterns observed between mono- and poly-specific intron splicing factors may reflect differences in their biochemical properties or molecular interaction networks.

**Figure 8.**
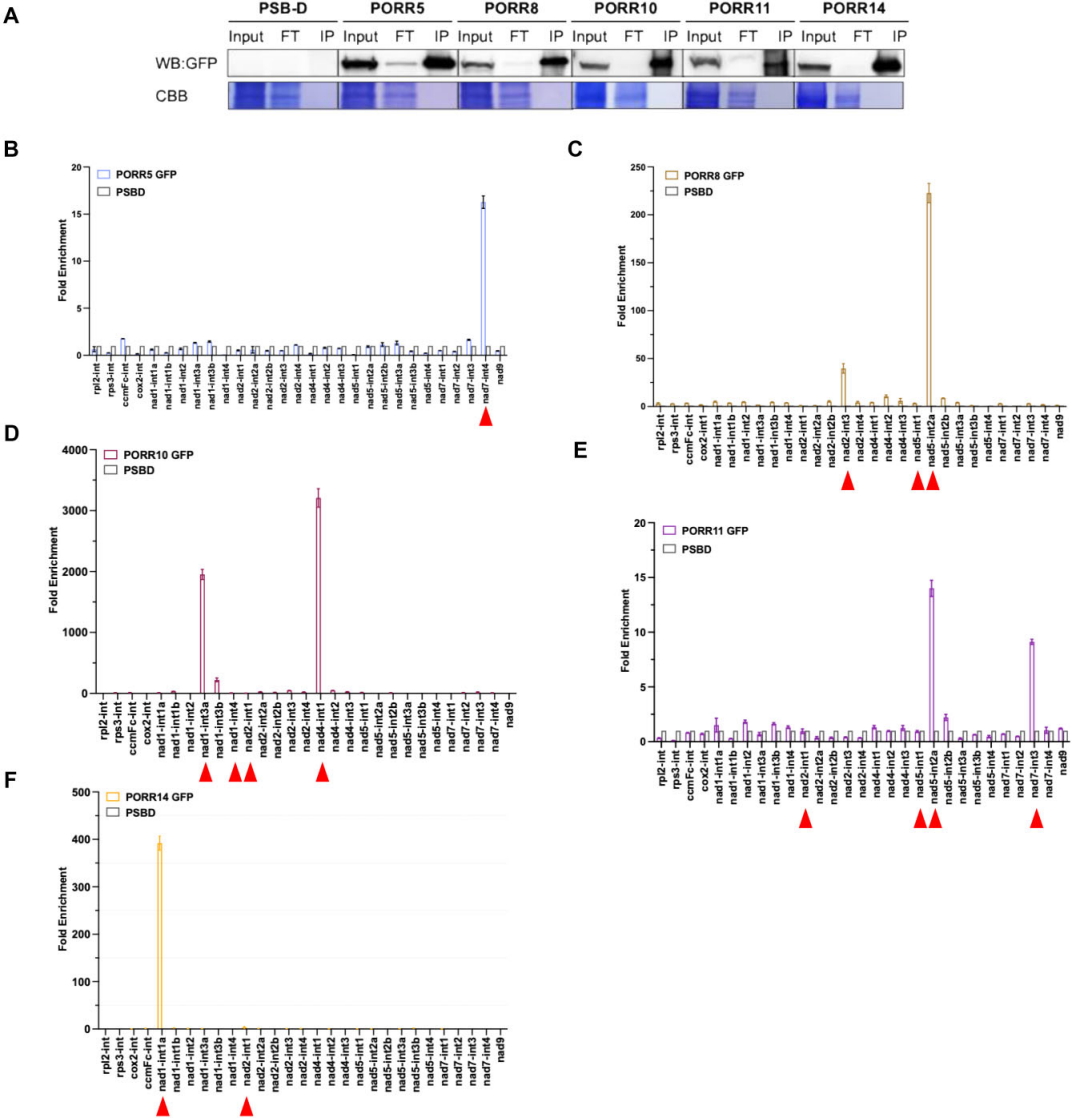
*In vivo* association analysis of PORR proteins with mitochondrial intron RNAs. (**A**) Immunodetection of immunoprecipitated PORR proteins. Total protein extracts from transgenic Arabidopsis cell lines expressing PORR-GFP fusions were subjected to immunoprecipitation using an anti-GFP antibody. Input (total protein extracts), flow-through, and immunoprecipitated fractions were analyzed by immunoblotting. Coomassie Brilliant Blue staining was used as a loading control. (**B**–**F**) RNA immunoprecipitation-RTqPCR analysis of RNA–PORR associations. RNA was extracted from the immunoprecipitates and analyzed by RT-qPCR using primer pairs targeting the intron/exon junctions of the indicated mitochondrial transcripts. RIP from untransformed Arabidopsis PSB-D lines (see “Materials and methods” section) served as a negative control. Panels (B) through (F) correspond to the results for PORR5, PORR8, PORR10, PORR11, and PORR14, respectively. For trans-spliced intron targets, the introns were annotated by their 5′ and 3′ halves, labeled as “a” and “b.” The data shown were generated from two biological replicates and three technical replicates.

PORR domain proteins have been shown to associate with RNA *in vivo* [30]. To investigate this further, we performed RNA immunoprecipitation assays followed by reverse transcription and quantitative PCR (RIP-RTqPCR) analysis. The five PORR proteins analyzed in this study were immunoprecipitated from the extracts of transformed Arabidopsis PSB-D cells, and the co-enriched RNAs were extracted from the immunoprecipitates (Fig. 8A). The extracted RNAs were then reverse transcribed and subjected to qPCR analysis, using primer pairs targeting the intron-exon junctions of mitochondrial precursor transcripts. The results revealed a significant and specific enrichment of PORR5 with *nad7* intron 4 (Fig. 8B). Similarly, strong enrichment was observed for the 5′ portion of the trans-spliced *nad5* intron 2 (*nad5*-int 2a) in PORR8 RNA immunoprecipitations, with a less prominent enrichment also detected for *nad2* intron 3 (Fig. 8C). For PORR10, the 5′ portion of the trans-spliced *nad1* intron 3 (*nad1*-int 3a) and *nad4* intron 1 showed high co-purification (Fig. 8D). Interestingly, *nad1* intron 4 and *nad2* intron 1 were not enriched in PORR10 coimmunoprecipitations, despite the fact that these introns require PORR10 for their splicing (Figs 3C and 8D). This suggests that PORR10 may influence the splicing of *nad1* intron 4 and *nad2* intron 1 indirectly or that its affinity for these introns is too low to be detected in our assay. For PORR11, the 5′ portion of the trans-spliced *nad5* intron 2 (*nad5*-int 2a) and *nad7* intron 3 coimmunoprecipitated with the protein, while *nad2* intron 1 and *nad5* intron 1 did not, despite the fact that their splicing requires the presence of PORR11 (Figs 3D and 8E). The 5′ portion of the trans-spliced *nad1* intron 1 (*nad1*-int 1a) was enriched in PORR14 co-immunoprecipitations, unlike *nad2* intron 1 (Fig. 8F). In summary, the five novel PORR proteins associate *in vivo* with most of the intron RNAs whose splicing they facilitate, further reinforcing their direct involvement in the splicing of these introns.

### PORR proteins bind to distinct intronic RNA regions

**Figure 9.**
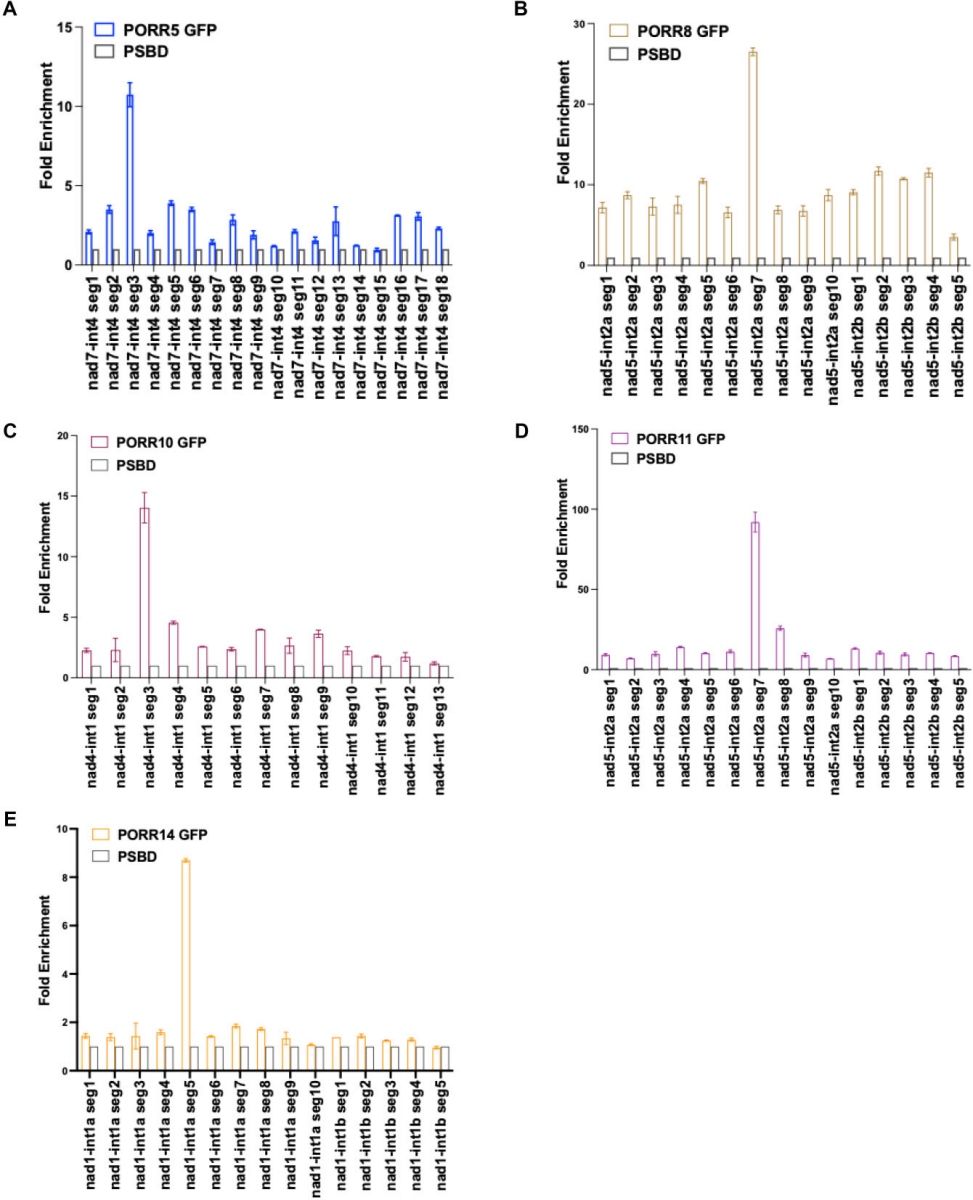
PORR proteins preferentially bind to 5′ regions of their intron targets. To precisely map the intronic regions bound by PORR proteins, extracts were treated with RNase I prior to immunoprecipitation. Protein-protected intronic RNA fragments were subsequently analyzed by RT-qPCR using overlapping amplicons (designated as segments, “seg”). Panels (**A**–**E**) show the mapping results for intron targets of PORR5, PORR8, PORR10, PORR11, and PORR14, respectively. Data represent the averages of two biological replicates and three technical replicates.

**Figure 10.**
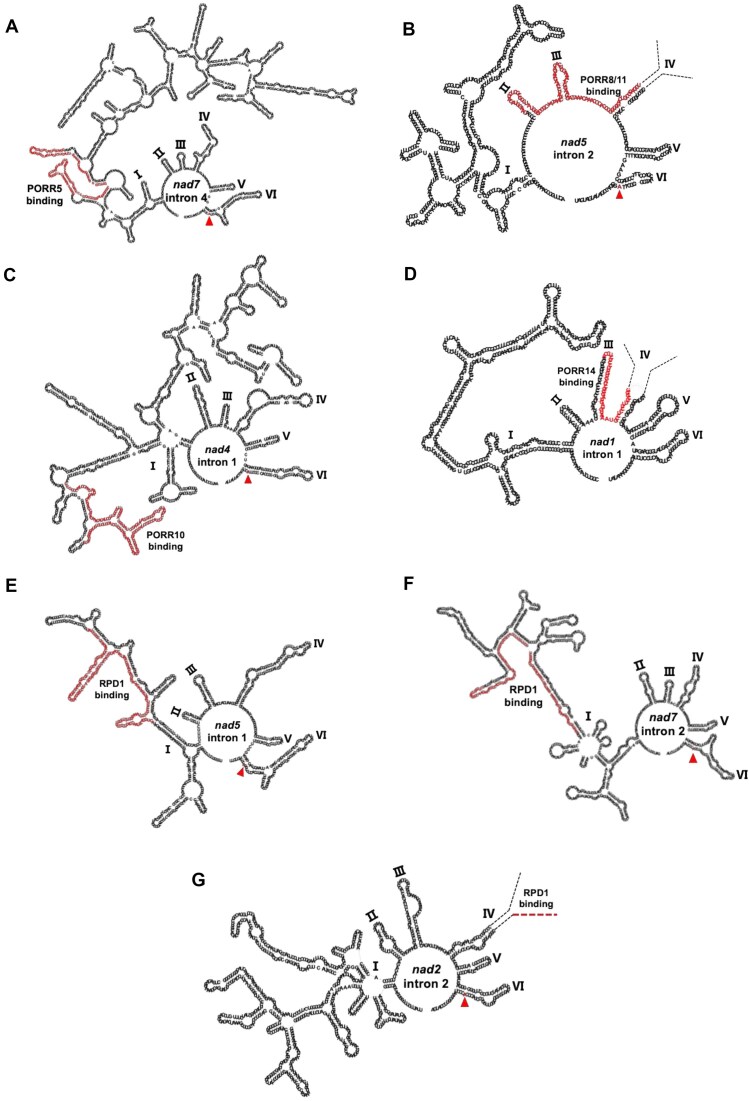
Secondary structure models of Arabidopsis mitochondrial introns showing PORR protein binding regions. The secondary structures of mitochondrial introns were modeled based on published group II intron RNA structures [45] and computational predictions generated using the MFOLD algorithm [46] and visualized using RNA Canvas [47]. Trans-spliced group II introns (*nad5* intron 2, *nad1* intron 1, and *nad2* intron 2) exhibit bipartite structures, with fragmentation sites located within DIV (indicated by dashed lines). The predicted bulged adenosine (bulged A), which serves as the intron branch point, is highlighted with a red triangle. Regions bound by PORR proteins are indicated in red. Panels (**A**–**D**) illustrate the binding sites for PORR5, PORR8, PORR11, PORR10, and PORR14, respectively, while panels (**E**) to (**G**) show the binding sites for RPD1.

Our previous study demonstrated that the RPD1 PORR protein binds to different regions within its numerous intron targets, suggesting variability in the role that this PORR protein plays in intron splicing [30]. To further investigate the nature of PORR-RNA interactions, we mapped the intronic binding sites of the five PORR proteins analyzed in this study using RIP-RTqPCR. This analysis was performed on RNase I-treated protein extracts using a series of overlapping primer pairs that targeted introns in ∼50-base pair segments. To better interpret the obtained results, the most enriched segments—corresponding to the *in vivo* RNA binding sites of the different PORR proteins—were mapped onto the predicted 2D folding structures of the respective introns (Figs 9 and 10). This approach revealed that several PORR proteins preferentially bind to domain I (DI) segments, including PORR5 in *nad7* intron 4, PORR10 in *nad4* intron 1, and RPD1 in *nad5* intron 1 and *nad7* intron 2 (Fig. 10). Additionally, the analysis identified several cases of binding to the 3′ regions of the 5′ half of trans-spliced introns. Specifically, PORR8 and PORR11 appeared to target the same region of *nad5* intron 2, spanning domains II, III, and the 5′ half of domain IV (DIV). Similarly, PORR14 binds to domain III and the 5′ half of DIV in *nad1* intron 1. Furthermore, RPD1 binds to the beginning of the 3′ half of the trans-spliced *nad2* intron 2, demonstrating that its binding site varies depending on the intron target. However, in some cases, no distinct binding regions were identified using our RNase I mapping approach, despite the fact that these introns were co-immunoprecipitated with PORR proteins (Fig. 8). This applies to *nad1* intron 3, a genetic target of PORR10, and *nad5* intron 2, whose splicing is facilitated by PORR11. In both cases, all defined RNA segments along these introns showed specific co-enrichment with their respective PORR proteins (Supplementary Fig. S5).

## Discussion

### Depending on the intron they target, plant mitochondrial PORR proteins associate with different intronic domains

Although sequence conservation among group II introns is minimal, their structural conservation is remarkable [36]. Group II introns adopt a characteristic architecture comprising six stem-loop domains (DI to DVI), with each domain playing a specific role in the splicing reaction. DI is the largest and most divergent domain at the sequence level; however, it acts as a scaffold for folding the other intron domains [37, 38] and properly positions the 5′ and 3′ exons in the reactive center for catalysis via its exon-binding site sequences. DII and DIII are significantly smaller but cooperate to organize the catalytic core and enhance splicing efficiency. DIV is highly variable in size and organization across group II introns. In some cases, it is split into two halves and carried by separate transcripts. DV is the most conserved domain, typically forming a 34-bp stem-loop structure. In the three-dimensional conformation, it integrates into the intron center, interacting with DI to organize the catalytic core and binding the two Mg^2+^ ions essential for catalysis. DVI is also short and generally harbors a branch-point adenosine that initiates splicing. Recent structural studies have revealed that DVI rotates after the first splicing step to pull the lariat intron out of the active site while simultaneously aligning the 3′ splice site for exon ligation [39]. Despite being classified as self-splicing introns, group II introns rarely splice autonomously *in vivo* and instead rely on protein cofactors, including maturases, which are often encoded within DIV. Structural data indicate that bacterial maturases play an allosteric role in the splicing reaction by stabilizing the branch-point adenosine near the 5′ splice site [39, 40]. In addition to maturases, numerous other protein factors, including PORR proteins, as demonstrated in this study, assist in intron splicing in plant mitochondria and plastids [20, 41]. Some plant mitochondrial group II introns require up to 10 different proteins for excision, always including a mitochondria- or nuclear-encoded maturase-like protein [20]. While their precise function remains unclear, it has been proposed that they either aid in proper intron folding or prevent the formation of non-productive structures [42]. Identifying the RNA binding sites of these splicing factors could provide insights into their function. Since very few of these factors have been analyzed in this regard [43], we thought that identifying the intron binding sites of a nearly complete protein family of splicing factors, such as the PORR proteins analyzed here, could provide new insights into how they facilitate splicing. As said, our genetic analysis demonstrates that PORR proteins participate in the splicing of 1 to 4 introns, with varying degrees of importance (Fig. 3). RIP-RTqPCR analysis revealed *in vivo* association of PORR proteins with all introns whose splicing efficiency is significantly reduced in their absence, suggesting a direct role for these proteins in the splicing of the concerned introns (Fig. 8). Interestingly, four out of the five PORR proteins analyzed were found required for the splicing of three different trans-spliced introns, suggesting a broad role for PORR proteins in trans-spliced intron splicing. RNase treatment prior to RIP-RTqPCR analysis allowed us to refine the intronic regions to which PORR proteins bind (Fig. 9). By positioning these binding sites onto the predicted secondary structures of these introns—along with that of the previously characterized RPD1 PORR protein [30]—we found that PORR5, PORR10, and RPD1 associate with specific regions within DI of their respective target introns. In contrast, PORR8 and PORR11 bind to a segment encompassing domains II and III (DII, DIII) as well as the 5′ half of DIV of *nad5* intron 2, while PORR14 associates with a region spanning DIII and extending into the 5′ half of DIV of *nad1* intron 1 (Fig. 10).

This analysis highlights the versatility of the PORR domain in accommodating various RNA target sites. Unlike helical repeat proteins such as PPR or mTERF factors, which bind RNA in a sequence-specific manner, the lack of repeated motifs in PORR proteins suggests that they likely recognize secondary or tertiary RNA structures. This is particularly evident in the case of RPD1, which binds to DI in two of its target introns but to DIV in another (Fig. 10). The observation that several PORR proteins (PORR8, PORR11, PORR14, and RPD1) bind to regions comprising the 5′ half of several trans-spliced introns suggests a potential role of PORR proteins in joining intronic halves to reconstitute functional trans-spliced introns. It would be particularly interesting to explore the potential functional collaboration between PORR8 and PORR11, as they share the same binding domain in the distal region of the 5′ half of *nad5* intron 2. As indicated, group II intron folding is thought to begin with DI, which serves as a scaffold for other domains to assemble into a catalytically active structure [37, 38]. The frequent and extensive sequence variations observed in DI (Fig. 10) suggest that compensatory protein cofactors may be required to stabilize its folding and maintain a functional catalytic core despite these modifications. Based on our findings, we propose that PORR5, PORR10, and RPD1 function as DI-specific RNA chaperones, either stabilizing DI structures in a catalytically competent conformation or preventing non-productive interactions between DI subdomains. To advance our understanding of how PORR and other organellar splicing factors promote group II intron splicing, high-resolution structural studies of intron-protein ribonucleoprotein complexes have now become essential. However, the large size of plant organellar introns and the large number of required splicing factors (often exceeding 10 per intron) pose significant challenges for structural characterization.

### Impaired complex I biogenesis results in lower Nad9 protein accumulation

Mitochondrial complex I (or NADH-ubiquinone oxidoreductase) is the first enzyme of the oxidative phosphorylation system and is the major entry point for electrons from NADH into the respiratory chain [44]. Complex I is an L-shaped multiprotein complex, with a hydrophobic membrane arm embedded in the inner mitochondrial membrane and a hydrophilic peripheral arm that extends into the mitochondrial matrix. Nine complex I subunits, referred to as Nad (NADH dehydrogenase) subunits, are encoded in the mitochondrial genome and located within the membrane arm of the complex.

Interestingly, our study revealed varying effects on the accumulation of the Nad9 subunit across the five *porr* mutants. While *porr10* and *porr14* mutants maintain Nad9 levels comparable to the wild type, *porr5*, *porr8*, and *porr11* mutants exhibit a significant reduction in Nad9 protein accumulation (Fig. 6). Transcriptome data suggest that this reduction is not caused by defects in *nad9* mRNA production, as *nad9* transcripts accumulate at normal levels in the mutants (Supplementary Fig. S2). None of the five PORR proteins were found to associate with *nad9* mRNA (Fig. 8), ruling out their role in regulating *nad9* translation. It rather suggests that the reduced levels in Nad9 protein are likely due to post-translational mechanisms, potentially through a negative feedback effect. These observations align with previous findings showing that defects in *nad2* or *nad7* protein production reduce Nad9 levels, while defects in other subunits, such as *nad1* or *nad5*, do not (summarized in Supplementary Table S1). This suggests that dysfunction in specific CI subunits triggers a negative feedback mechanism regulating Nad9 protein accumulation, likely associated with the stepwise assembly process of CI. In Arabidopsis, mitochondrial CI assembles through the formation of the matrix arm (comprising the N and Q modules) and the membrane arm (comprising the P_P_ and P_D_ modules). Nad7 and Nad9 are key components of the Q module and integrate early in it. In contrast, the P_P_ module, which includes subunits such as Nad2 and Nad1, is proposed to assemble into a CI intermediate of ∼450 kDa that subsequently associates with the matrix arm [31]. Nad2 associates early during P_P_ module formation to produce a 200 kDa assembly intermediate, whereas Nad1 participates in the final step, converting a 400 kDa assembly intermediate into a 450 kDa one. Our results suggest that the step at which the assembly process of the P_P_ module is interrupted may lead to secondary defects that result in the degradation of Q module components, including Nad9 and Nad7. According to the assembly model, the absence of Nad2 production blocks P_P_ module assembly at its initial stage, thereby impeding the production of subsequent intermediates (400 and 450 kDa) that may protect the matrix arm (and consequently Nad9 and Nad7) from degradation through physical association. Conversely, a shortage in Nad1 production allows the assembly to progress up to the 400 kDa intermediate, which may associate with the Q module, providing protection against the degradation of Nad9 and Nad7. This hypothesis may explain why the accumulation of the Nad9 and Nad7 proteins is consistently and significantly reduced in plant respiratory mutants affected by a lack of Nad2 production, but not in those lacking Nad1, as observed in the *porr8* and *porr14* mutants (Fig. 6). Furthermore, our results reveal that the lack Nad7 production negatively impacts Nad9 accumulation (see results for *porr5* and *porr11* mutants in Figs. 3 and 6), suggesting that Nad7 and Nad9 production are interdependent and require association with the membrane arm for stable accumulation. The degradation of non-assembled matrix arm modules may serve as regulatory mechanism to prevent unproductive reduction of NADH and the generation of harmful, non-channeled electrons in the mitochondrial matrix.

## Supplementary Material

gkaf781_Supplemental_File

## Data Availability

Sequence data from this article can be found in the GenBank/EMBL data libraries under accession numbers RPD1, AT4G33495; PORR5, AT5G45790; PORR8, AT2G31290; PORR10, AT4G08940; PORR11, AT3G63090; and PORR14, AT1G06440.
