# Supplementary material for: Plant mitochondrial PORR proteins facilitate group II intron splicing by binding to distinct regions within their target introns
Source: Nucleic Acids Res. 2025 Aug 18;53(15):gkaf781. doi: 10.1093/nar/gkaf781 (PMC12359027; doi:10.1093/nar/gkaf781)
Supplement: gkaf781_Supplemental_File [file gkaf781_supplemental_file.pdf]

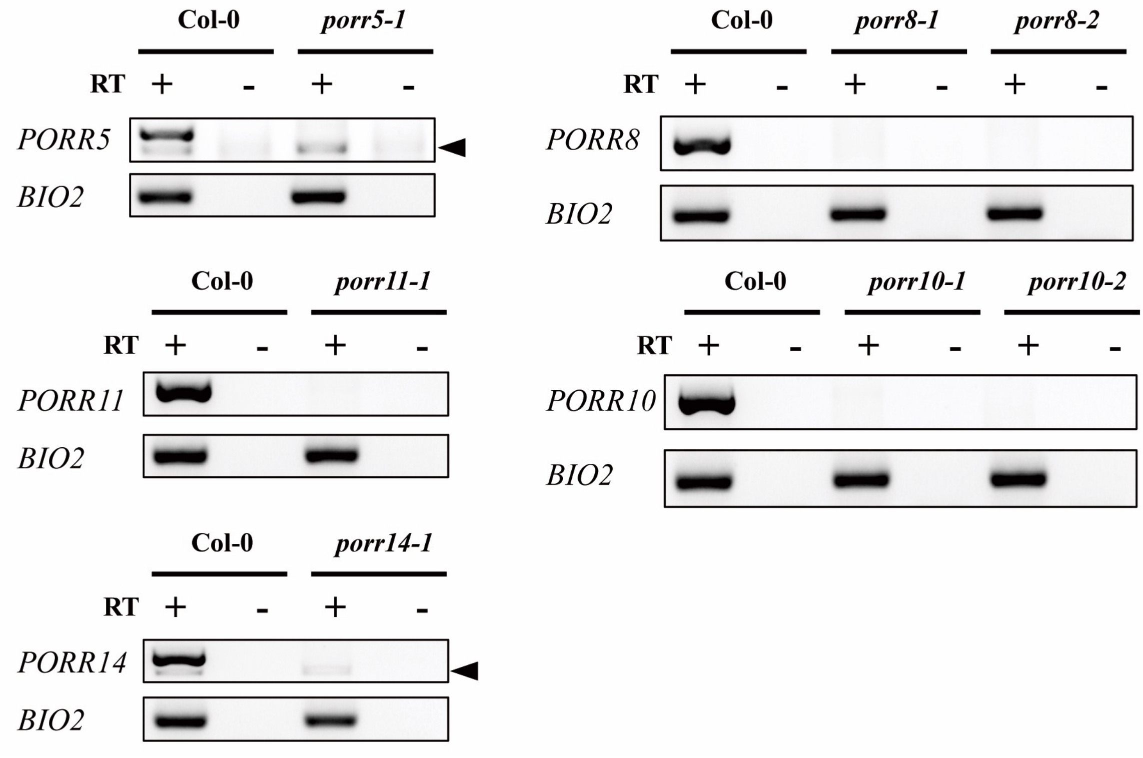

Supplemental Figure 1. RT-PCR analysis of *PORR* transcripts in wild-type and *porr* mutants. Total cDNA was synthesized from the indicated genotypes, and corresponding *PORR* transcripts were amplified by PCR using primers flanking the T-DNA insertion sites. PCR products were separated by agarose gel electrophoresis. To verify cDNA synthesis in all analyzed samples, *BIO2* cDNA was separately amplified from each genotype. Reverse transcriptase was either included (RT+) or omitted (RT-) during cDNA synthesis as indicated. Non-specific bands are marked by shaded triangles.

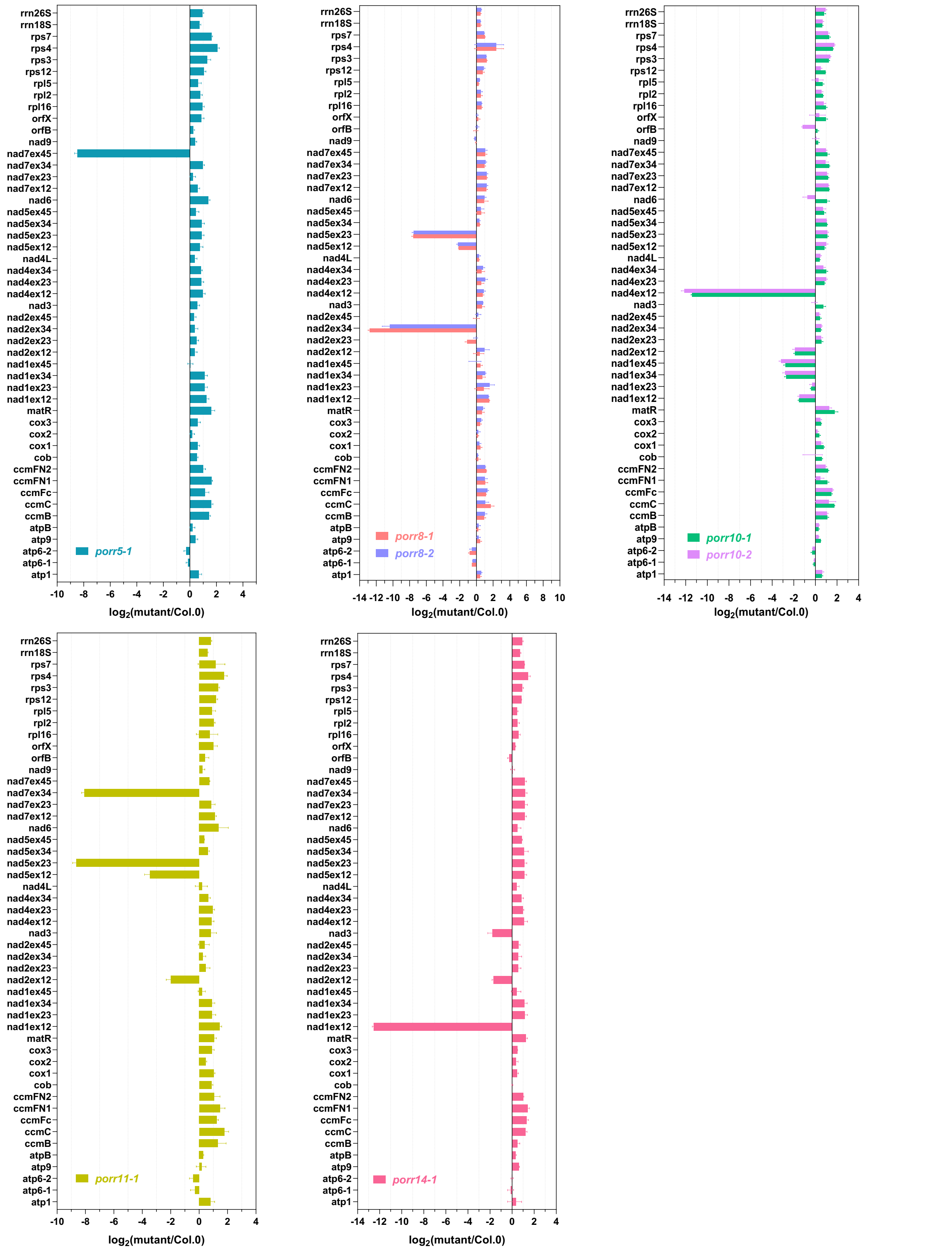

Supplemental Figure 2. RT-qPCR measuring the steady state levels of mature mitochondrial mRNAs in Col-0 and *porr* plants. A single PCR was performed for mRNAs containing only one exon, while PCR were conducted using primer pairs across introns for intron-containing mRNAs. Two biological replicates and three technical replicates were used per genotype; standard errors are indicated.

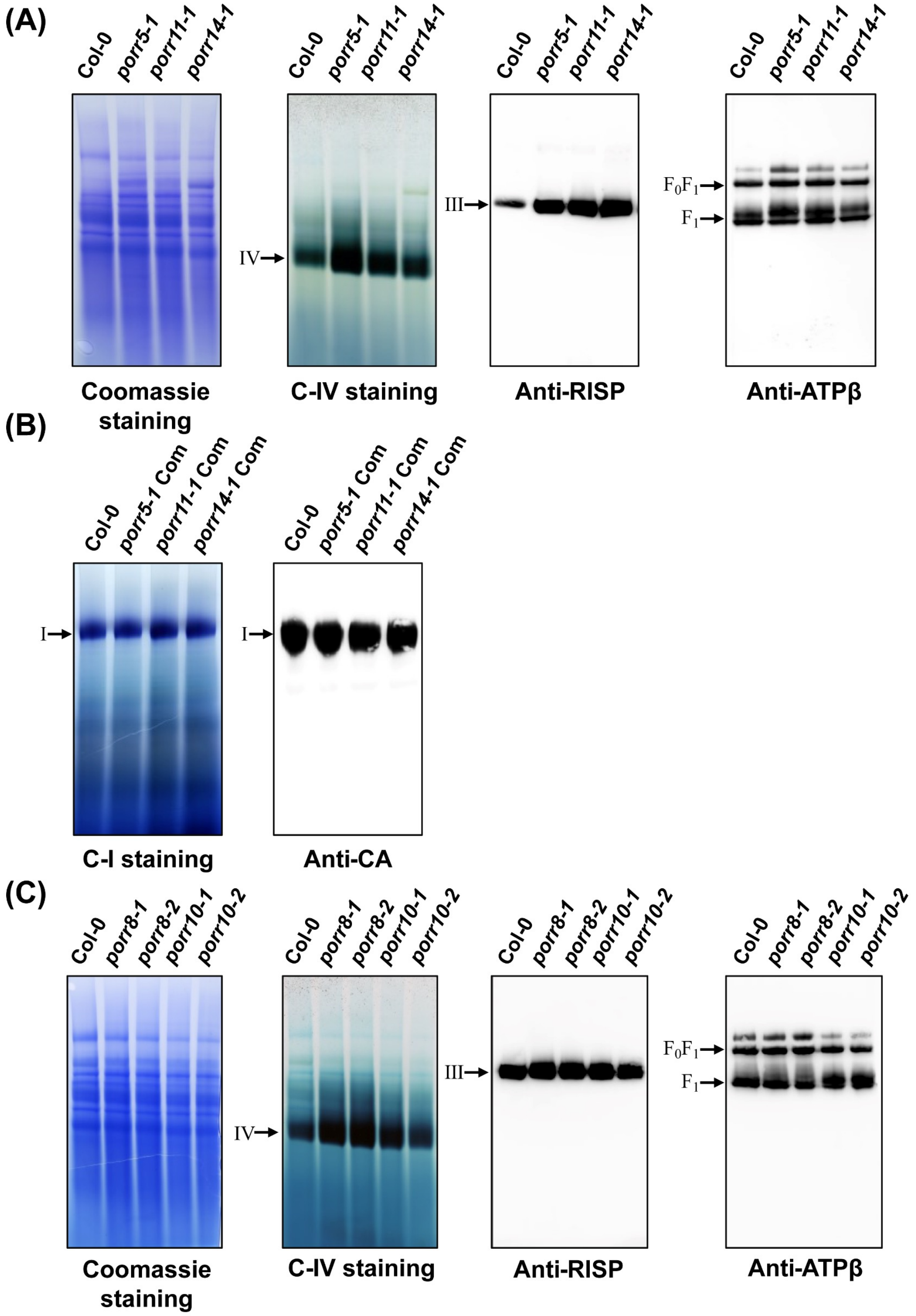

Supplemental Figure 3. BN-PAGE and immunoblot analysis of mitochondrial complexes in *porr* (A and C) and functionally-complemented mutants (B). Crude mitochondrial extracts prepared from wild-type and *porr* mutants or complemented plants (Com) were separated on BN-PAGE gels and stained with Coomassie blue. In-gel staining revealing the NADH dehydrogenase activity of complex I and cytochrome *c* oxidase activity of complex IV are also presented. For immunoblot analysis, BN-PAGE gels were transferred onto PVDF membranes and probed with anti-RISP and anti-ATPase-β antibodies to assess steady-state levels of complex III and complex IV, respectively.

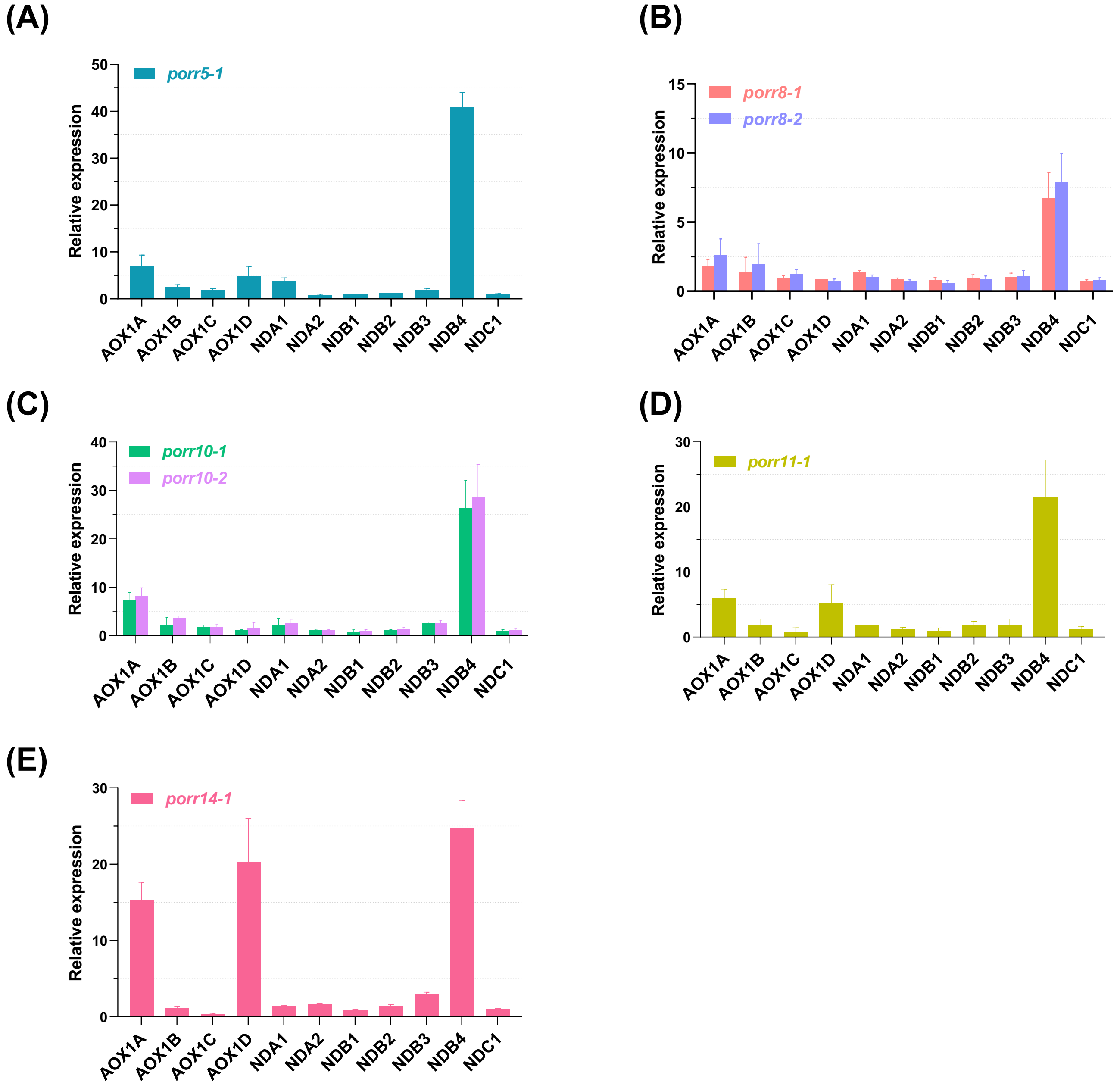

Supplemental Figure 4. The alternative respiratory pathway is induced in the five *porr* mutant plants. The relative accumulation levels of alternative oxidase (AOX) and NADH dehydrogenase (NDA, NDB and NDC) transcripts was measured by RT-qPCR in wild-type and *porr* mutants. Two biological repeats and three technical repeats were performed for each genotype in this analysis. standard errors are indicated.

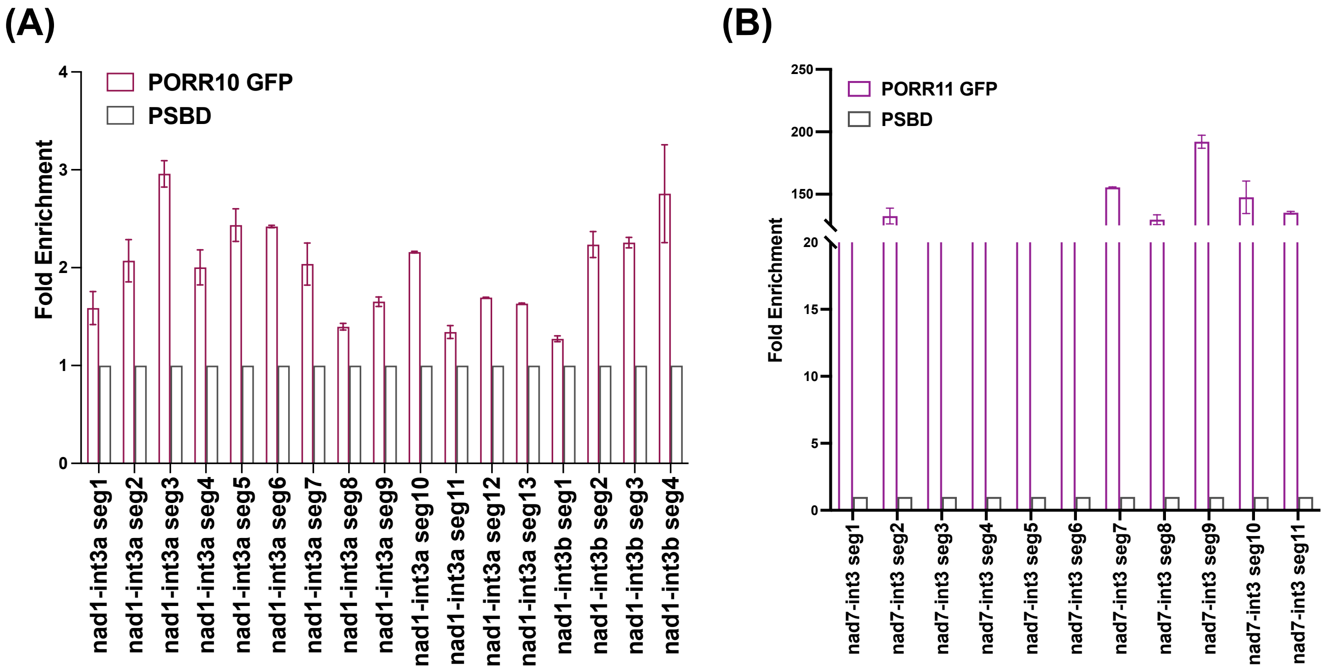

Supplemental Figure 5. The precise binding sites of PORR10 and PORR11 within *nad1* intron 3 and *nad7* intron 3 could not be identified. Panels A and B depict co-enrichment of PORR10 with the *nad1* intron 3 segments and PORR11 with the *nad7* intron 3 segments, respectively.

Supplemental Table S1. Report on the steady-state levels of Nad9 protein in published mutants with defects in various complex I subunits.

Gene ID	Gene symbol	Mito subunit affected	Complex I module affected	Nad9 protein abundance	Reference
AT3G48250	BIR6	Nad7	Q	reduce	(Koprivova *et al*, 2010)
AT5G64320	MTL1	Nad7	Q	reduce	(Haili *et al*, 2016)
AT5G45790	PORR5	Nad7	Q	reduce	This study
AT2G31290	PORR8	Nad2, Nad5	Pp and P_D	reduce	This study
AT3G63090	PORR11	Nad7, Nad5	Pp and P_D	reduce	This study
AT3G09060	EMS1	Nad2	Pp	reduce	(Wang *et al*, 2023)
AT2G02150	MTSF3	Nad2	Pp	reduce	(Wang *et al*, 2022)
AT5G60870	RUG3	Nad2	Pp	reduce	(Kuhn *et al*, 2011)
AT1G74350	nMAT4	Nad1	Pp	non changed	(Cohen *et al*, 2014)
AT1G52620	PPR19 (MTSF2)	Nad1	Pp	non changed	(Lee *et al*, 2017)
AT5G04050	nMAT3	Nad1	Pp	non changed	(Shevtsov-Tal *et al*, 2021)
AT4G20090	MSP1	Nad1	Pp	slightly increase	(Best *et al*, 2023)
AT1G06440	PORR14	Nad1	Pp	slightly increase	This study
AT3G22450	uL18-L1	Nad5	P_D	non changed	(Wang *et al*, 2020)
AT4G08940	PORR10	Nad1, Nad4	Pp and P_D	non changed	This study
AT1G30010	nMAT1	Nad1, Nad4	Pp and P_D	non changed	(Keren *et al*, 2012)

Best C, Mizrahi R, Edris R, Tang H, Zer H, Colas des Francs-Small C, Finkel OM, Zhu H, Small ID, Ostersetzer-Biran O (2023) MSP1 encodes an essential RNA-binding pentatricopeptide repeat factor required for *nad1* maturation and complex I biogenesis in Arabidopsis mitochondria. *New Phytol* 238: 2375-2392

Cohen S, Zmudjak M, Colas des Francs-Small C, Malik S, Shaya F, Keren I, Belausov E, Many Y, Brown GG, Small I *et al* (2014) nMAT4, a maturase factor required for *nad1* pre-mRNA processing and maturation, is essential for holocomplex I biogenesis in Arabidopsis mitochondria. *Plant J* 78: 253-268

Haili N, Planchard N, Arnal N, Quadrado M, Vrielynck N, Dahan J, des Francs-Small CC, Mireau H (2016) The MTL1 Pentatricopeptide Repeat Protein Is Required for Both Translation and Splicing of the Mitochondrial *NADH DEHYDROGENASE SUBUNIT7* mRNA in Arabidopsis. *Plant Physiol* 170: 354-366

Keren I, Tal L, des Francs-Small CC, Araujo WL, Shevtsov S, Shaya F, Fernie AR, Small I, Ostersetzer-Biran O (2012) nMAT1, a nuclear-encoded maturase involved in the trans-splicing of *nad1* intron 1, is essential for mitochondrial complex I assembly and function. *Plant J* 71: 413-426

Koprivova A, des Francs-Small CC, Calder G, Mugford ST, Tanz S, Lee BR, Zechmann B, Small I, Kopriva S (2010) Identification of a pentatricopeptide repeat protein implicated in splicing of intron 1 of mitochondrial *nad7* transcripts. *J Biol Chem* 285: 32192-32199

Kuhn K, Carrie C, Giraud E, Wang Y, Meyer EH, Narsai R, des Francs-Small CC, Zhang B, Murcha MW, Whelan J (2011) The RCC1 family protein RUG3 is required for splicing of *nad2* and complex I biogenesis in mitochondria of Arabidopsis thaliana. *Plant J* 67: 1067-1080

Lee K, Han JH, Park YI, Colas des Francs-Small C, Small I, Kang H (2017) The mitochondrial pentatricopeptide repeat protein PPR19 is involved in the stabilization of *NADH dehydrogenase 1* transcripts and is crucial for mitochondrial function and Arabidopsis thaliana development. *New Phytol* 215: 202-216

Shevtsov-Tal S, Best C, Matan R, Chandran SA, Brown GG, Ostersetzer-Biran O (2021) nMAT3 is an essential maturase splicing factor required for holo-complex I biogenesis and embryo development in *Arabidopsis thaliana* plants. *Plant J* 106: 1128-1147

Wang C, Blondel L, Quadrado M, Dargel-Graffin C, Mireau H (2022) Pentatricopeptide repeat protein MTSF3 ensures mitochondrial RNA stability and embryogenesis. *Plant Physiol* 190: 669-681

Wang C, Fourdin R, Quadrado M, Dargel-Graffin C, Tolleter D, Macherel D, Mireau H (2020) Rerouting of ribosomal proteins into splicing in plant organelles. *Proc. Natl. Acad. Sci. U. S. A.* 117: 29979-29987

Wang C, Quadrado M, Mireau H (2023) Interplay of endonucleolytic and exonucleolytic processing in the 3'-end formation of a mitochondrial *nad2* RNA precursor in Arabidopsis. *Nucleic Acids Res.* 51: 7619-7630

Supplementary Table S2. Oligonucleotides used in this study.

Oligonucleotides	Sequence 5'-3'	Experiment
PORR5-1	AGGTTGATGCTGTGAAGAGGG	Genotyping and RT-PCR
PORR5-2	CTGCAACAACACGCTTCTCG	
PORR8-2	TGCAGAGACAGCCAACTCAG	
PORR8-4	CGTTTGATTTGCAAGCTGTGG	
PORR8-9	ATGGCTATGACACGAGAGTT	
PORR10-1	ACATTTCCGATTCCTAATCCG	
PORR10-4	AACACAACCTGTGGATCTT	
PORR10-8	ATGGCTTGGCTTCGTTTCTT	
PORR11-1	AGCAGATAAAGGAGCAAAGGG	
PORR11-2	GGTAAACCGGTTAGCCTGAAG	
PORR11-8	ATGGTGCTCAGAAACTTAACC	
PORR14-3	TAATTCCGCAGAGAGTGTGGG	
PORR14-4	GAACTCGTGAGCAATCGCAA	
PORR14-8	ATGAGGTTCCTCCTCATGAG	
LBSALK2	GCTTTCTTCCCTTCCTTTCTC	
LB3SAIL	TAGCATCTGAATTTCATAACCAATCTCGATACAC	
BIO2-6	AGAAACGATTGGAGTAGAGATGA	
BIO2-8	AATTGTATCTCGCCAAGC	
GWPORR5-1	CAAAAAAGCAGGCTCTAATTATTTTCTGAATATTGGT	Complementation and GFP fusion
GWPORR5-2	CAAGAAAGCTGGGTCTTTCCCCAAAATTGGTAAGAC	
GWPORR8-1	CAAAAAAGCAGGCTCTGGTTTCACCGAGACTCAGGGC	
GWPORR8-2	CAAGAAAGCTGGGTCTTGGGTGTCACTGAAGAGAATCTGAT	
GWPORR10-1	CAAAAAAGCAGGCTCTTATCTTAGTAACAATCCATAC	
GWPORR10-2	CAAGAAAGCTGGGTCACCAACTTGCTCCATCTGCTG	
GWPORR11-1	CAAAAAAGCAGGCTCTATGTGTGCATGAACAGAAGTT	
GWPORR11-2	CAAGAAAGCTGGGTCATCACAGAGACGCTCTATCGG	
GWPORR14-1	CAAAAAAGCAGGCTCTTAATAAAAATTAGGGTTCTTT	
GWPORR14-2	CAAGAAAGCTGGGTCTTCTTGTTCAGACATACTAGT	
GWPORR5-3	CAAAAAAGCAGGCTCTATGTTCGAAAAATGTTTCCTT	
GWPORR8-3	CAAAAAAGCAGGCTCCATGGCTATGACACGAGAGTTA	
GWPORR10-3	CAAAAAAGCAGGCTCTATGGCTTGGCTTCGTTTCTTC	
GWPORR11-3	CAAAAAAGCAGGCTCTATGGTGCTCAGAAACTTAACC	
GWPORR14-3	CAAAAAAGCAGGCTCCATGAGGTTCCTCCTCATGAGA	
nad1e7	CATCACCCTAGCAAGCCTAAC	Probes preparation for RNA blots
nad1e8	TAAGGAAGCCATTGAAAGGTG	
nad2e1	GCAGAATTCGTTCGGATC	
nad2e8	TATGAACTGAGTGCCATTTGA	
nad4F	ATTCTATGTTTTTCCCGAAAGC	
nad4R	TGAAATTTGCCATGTTGCAC	
nad5 E7	AGAAGGAAGCGCTATAATGAC	
nad5 E6	TACTCACTATCAAAATGAAAG	
nad7F	ATGACGACTAGGAAAAGGCA	
nad7R	CTATCTATCCACCTCTCCAA	
nad9F	TTGGGTCATCTCAATGGGTT	
nad9R	CGATCGATATTTGCGGAGTT	

QMnad1exon1R	TTGCCATATCTTCGCTAGGTG	
nad1i1a	CTAGCCCTGTAAGCCCACAC	
NAD1exon2_R	GACCAATAGATACTTCATAAGAGACCA	
Qmnad1intron1R	CGTGCTCGTACGGTTCATAG	
QMnad1intron2F	GGTTGGGTTAGGGGAACATC	
QM1120F	TCTGCAGCTCAAATGGTCTC	
QMnad1exon3R	TCCGTTTGATCTCCCAGAAG	
nad1i3c	CTTCCGCCAACAGTGGACTA	
NAD1_exon4_R	AAAAGAGCAGACCCCATTGA	
QMnad1intron3R	GGGAGCTGTATGAGCGGTAA	
QMnad1exon5F	AGCCCGGGATCTTCTTGA	
QMnad1intron4R	ACGGAGCTGCATCCCTACT	
QM0285R	GCGAGCAGAAGCAAGGTTAT	
mito091R	CCCATTCCTAACCAGTGGAG	
QMnad2intron2F	CCCGATCCGATAGTTTACAA	
QMnad2exon2R	AATATTTGATCTTAGGTGCATTTTC	
nad2 int2b-1	AGTGGGAAGAAAAGGCTCGG	
nad2 ex3-1	GGGTAGCTCCAGTAGACCCA	
mito338F	GCGCAATAGAAAGGAATGCT	
QMnad2intron3R	GGCGAATTTCAAACTTGTGG	
mito337F	CTTATTCGTGGCAACCTTCC	
QM1320F	TATTTGTTCTTCGCCGCTTT	
mito186F	CCGTATGATGCGGAAGTCTC	
QMnad4exon2R	GAAAAACTGATATGCTGCCTTG	
QMnad4intron2F	GCGGAACGACCAGAAAAATA	
QM0580R	TGCTACCTCCAATTCCCTGT	
QMnad4intron3F	TCTAGCTTGGTTCGGAGAGC	
nad4exon4R	TGAAATTTGCCATGTTGCAC	RIP-qPCR
QMnad5exon2F	TGGACCAAGCTACTTATGGATG	
QMnad5intron1R	TTCGCAAATAGGTCCGACT	
QMnad5intron2F	GTACGATCGTGTCGGGTGA	
QMnad5exon2R	CTGGCTCTCGGGAGTCTCTT	
nad5 int2b-R	GTGTACAACAACCTTAACC	
nad5_exon3_F	TACCTAAACCAATCATCATATC	
nad5_exon3_R	GATATGATGATTGGTTTAGGTA	
nad5 int3a-F	TTATTCTCAAGTAAAGGGTG	
QMnad5exon4F	AACTCGGATTCGGCAAGAA	
QMnad5intron3R	GCCGTGTAATAGGCGACCA	
QM0060F	AACATTGCAAAGGCATAATGA	
QMnad5intron4R	CCTGTAAACCCCCATGATGT	
QMnad7intron1F	ACGGTTTTTAGGGGGATCTG	
nad7exon2R	AAGGTAAAGCTTGAAGATAAGTTTTGT	
mito128F	AGTGGGAGAGCCGTGTTATG	
QMnad7exon3R	TGGTACCTCGCAATTCAAAA	
mito131F	TAAAGTGAAGTGGTGGGCCT	
QM0510R	CATTGCACAATGATCCGAAG	
mito135F	CGGCCAAATGACTACAGGAT	
QMnad7exon5R	AGGTGCTTCAACTGCGGTAT	
mito170F	TTAGGAAGAGCCGTACGAGG	
QM0560R	CGCAATTCATCACCATTTTG	
QM090R	AGCCGAAGGTGAGTCTCGTA	
QMrps3intronR	TCTACGGCGGGGTCACTAT	
QM0160R	TGGGGGATTAATTGATTGGA	
mito037R	AGCAGTACGAGCTGAAAGGC	
QMccb452intronF	CCCGGATCGAATCAGAGTT	
QM180F	CACATGGAGGAGTGTGCATC	

Supplemental Table S3. List of antibodies used in this study.

Antibody	Host	Dilution	Source
AOX1a	Rabbit	1 : 1,000	Agrisera (AS04 054)
ATPβ	Chicken	1 : 5,000	Agrisera (AS05 085)
CA2	mouse	1 : 5,000	Sunderhaus et al., 2006
Cox2	Rabbit	1 : 1,000	Agrisera (AS04 053A)
CYT *c*	Rabbit	1 : 5,000	Agrisera (AS08 343A)
GFP (3H9)	Rat	1 : 1,000	Chromotek (3h9-20)
HA (clone 3F10)	Rat	1 : 500	Roche (11867423001)
Nad7	Rabbit	1 : 10,000	Pineau *et al*., 2008
Nad9	Rabbit	1 : 5,000	Lamattina L. *et al*., 1993
RISP	Rabbit	1 : 5,000	Chris C. *et al*., 2010
PORIN	Mouse	1 : 1,000	Taylor et al. 2003

Sunderhaus S, Dudkina NV, Jansch L, Klodmann J, Heinemeyer J, Perales M, Zabaleta E, Boekema EJ, Braun HP (2006) Carbonic anhydrase subunits form a matrix-exposed domain attached to the membrane arm of mitochondrial complex I in plants. J Biol Chem 281: 6482-6488

Pineau B, Layoune O, Danon A, De Paepe R (2008) L-galactono-1,4-lactone dehydrogenase is required for the accumulation of plant respiratory complex I. J Biol Chem 283: 32500-32505

Lamattina L, Gonzalez D, Gualberto J, Grienenberger JM (1993) Higher plant mitochondria encode an homologue of the nuclear-encoded 30-kDa subunit of bovine mitochondrial complex I. Eur J Biochem 217: 831-838

Carrie C, Giraud E, Duncan O, Xu L, Wang Y, Huang S, Clifton R, Murcha M, Filipovska A, Rackham O, Vrielink A, Whelan J (2010) Conserved and novel functions for Arabidopsis thaliana MIA40 in assembly of proteins in mitochondria and peroxisomes. J Biol Chem 285: 36138-36148

Taylor NL, Rudhe C, Hulett JM, Lithgow T, Glaser E, Day AD, Millar AH, Whelan J. 2003. Environmental stresses inhibit and stimulate different protein import pathways in plant mitochondria. FEBS Lett 547:125-130.
